# Quantification of Histone Deacetylase Isoforms in Human Frontal Cortex, Human Retina, and Mouse Brain

**DOI:** 10.1371/journal.pone.0126592

**Published:** 2015-05-11

**Authors:** Kyle W. Anderson, Junjun Chen, Meiyao Wang, Natalia Mast, Irina A. Pikuleva, Illarion V. Turko

**Affiliations:** 1 Institute for Bioscience and Biotechnology Research, Rockville, Maryland, United States of America; 2 Biomolecular Measurement Division, National Institute of Standards and Technology, Gaithersburg, Maryland, United States of America; 3 Department of Chemistry and Biochemistry, University of Maryland, College Park, Maryland, United States of America; 4 Department of Ophthalmology and Visual Sciences, Case Western Reserve University, Cleveland, Ohio, United States of America; Pacific Northwest National Laboratory, UNITED STATES

## Abstract

Histone deacetylase (HDAC) inhibition has promise as a therapy for Alzheimer’s disease (AD) and other neurodegenerative diseases. Currently, therapeutic HDAC inhibitors target many HDAC isoforms, a particularly detrimental approach when HDAC isoforms are known to have different and specialized functions. We have developed a multiple reaction monitoring (MRM) mass spectrometry assay using stable isotope-labeled QconCATs as internal standards to quantify HDAC isoforms. We further determined a quantitative pattern of specific HDACs expressed in various human and mouse neural tissues. In human AD frontal cortex, HDAC1,2 decreased 32%, HDAC5 increased 47%, and HDAC6 increased 31% in comparison to age-matched controls. Human neural retina concentrations of HDAC1, 2, HDAC5, HDAC6, and HDAC7 decreased in age-related macular degeneration (AMD)-affected donors and exhibited a greater decrease in AD-affected donors in comparison to age-matched control neural retinas. Additionally, HDAC concentrations were measured in whole hemisphere of brain of 5XFAD mice, a model of β-amyloid deposition, to assess similarity to AD in human frontal cortex. HDAC profiles of human frontal cortex and mouse hemisphere had noticeable differences and relatively high concentrations of HDAC3 and HDAC4 in mice, which were undetectable in humans. Our method for quantification of HDAC isoforms is a practical and efficient technique to quantify isoforms in various tissues and diseases. Changes in HDAC concentrations reported herein contribute to the understanding of the pathology of neurodegeneration.

## Introduction

Alzheimer’s disease (AD) is the leading neurodegenerative disorder in the U.S. and affects approximately 13% of people over the age of 65 years and 45% over 85 [1]. AD pathogenesis is still largely unknown but it is suggested that changes may occur as early as 20 years prior to the development of symptoms and disease indicators [1], such as dementia, β-amyloid plaques, and tau aggregates. These early changes are likely present on the epigenetic level where gene expression is controlled. One method of controlling gene expression is through modulation of chromatin density by histone modifications. Histone post-translational modifications (PTMs) alter chromatin structure by dictating histone-DNA and inter-nucleosome interactions [2]. Changes in chromatin structure regulation have been linked to neurodegeneration and AD, giving credence to focusing on epigenetic targets for treatment of neurodegeneration [3–5].

One of the most common PTMs in histones is acetylation. Removal of acetyl groups from histone tails is chiefly catalyzed by histone deacetylases (HDACs). HDACs are categorized into eleven main isoforms, which are further broken into thirty-eight sequence variants by truncations, deletions, and substitutions of the canonical sequence [6]. Several HDACs have been linked to memory impairment and dementia [7,8], a hallmark of AD, and it has been demonstrated that global deacetylation of histones and overall activity of HDACs is increased in AD [9]. In addition to histones, HDACs are also known to modify over 50 non-histone proteins [9]. The majority of information on effects of HDAC isoforms comes from overexpression and knockouts of HDACs in murine models of AD. While mice may be more practical for neurodegeneration research involving manipulation of HDACs, it is still a non-human model for familial, early-onset AD. Most AD cases are considered sporadic or late onset, and while they may have the same key characteristics like dementia and protein aggregation, pathogenesis may vary from familial AD [10]. A connection between HDACs and AD has been established in mouse models; however, little information exists on changes in specific isoforms and the significance of their effects on AD pathology in humans.

HDAC inhibitors (HDIs) have shown improvement of AD-related symptoms; however, these are broad class HDIs, which do not target specific isoforms. If a long-term regimen of broad class HDIs were prescribed to prevent or stop the progression of AD, there may be deleterious side effects in other HDAC-associated pathways. For example, HDIs in mouse models have demonstrated improvement in memory [7,11,12], yet deficiency of either HDAC4 or HDAC5 impairs memory [13,14]. Specific knowledge of isoforms directly related to AD is imperative for treatment specificity and safety [15].

Various methods for assessment of HDAC levels have been reported, including quantitative real-time polymerase chain reaction [16,17], *in situ* hybridization [18], Western blotting [16,17], and immunohistochemical staining [19]. However, these semi-quantitative methods are unable to provide absolute quantitative data on the protein level of HDAC isoforms and isoform-specific quantification of HDACs remains elusive. Mass spectrometry (MS) provides the potential to perform targeted absolute quantification of protein isoforms. Synthetic peptides and full-length proteins labeled with stable isotopes are commonly used in MS-based protein quantification. Due to the large number of HDAC isoforms, it is less practical to use synthetic peptides or express full-length protein for each isoform. An alternative is performing quantitative measurements using quantification concatamers (QconCATs) as internal standards [20,21]. QconCATs are standard proteins comprised of proteolytic peptides used for quantification and they may include the respective natural flanking sequences from targeted proteins. Our previous work has shown applicability of QconCATs for measurement of abundant proteins in neural tissues, such as amyloid precursor protein [22], apolipoprotein E [23], clusterin [24], PICALM [25], and ubquiting carboxy-terminal hydrolase L1 [26]. QconCATs used in this study exceed past number of proteins measured and cover 25 sequence variants of HDACs, proteins with significantly lower abundance than those previously reported.

The goal of the present study was to determine whether multiplexing QconCAT technology could be applied to quantification of HDAC isoforms in different neural tissues in normal and disease state. Using QconCAT technology [20], three ^15^N-labeled standard proteins were produced to measure all selected HDAC variants. Human and mouse brain and human retina samples were supplemented with these standard proteins and protocols for sample processing were optimized and analytically characterized. Measurements in human frontal cortex and human retina give insight into abundance of particular isoforms in different neural tissues in normal and disease states. Mouse brain, a common model for neurodegenerative phenotypes, shows HDAC profiles in comparison to human tissue. Quantification of HDAC isoforms in AD-affected human brain and age-matched controls also contributes to our knowledge of disease-associated isoforms that may be of value for HDIs therapies for AD. Furthermore, the developed analytical approach is broadly applicable to quantitative analysis of HDAC variants in various tissues and disease models.

## Methods

### Materials

The DC Protein Assay kit was purchased from Bio-Rad Laboratories (Hercules, CA). Ammonium chloride (^15^N, 99%) was purchased from Cambridge Isotope Laboratories (Andover, MA). Sequencing grade modified trypsin, from Promega Corp. (Madison, WI), was used for QconCAT characterization experiments. Trypsin T0303 Type IX-S from porcine pancreas, purchased from Sigma-Aldrich (St. Louis, MO), was used for biological sample preparations. All other chemicals, including HDAC6 human recombinant protein, were purchased from Sigma-Aldrich (St. Louis, MO).

### Expression, purification, and characterization of ^15^N-labeled QconCATs

Tryptic peptides for all HDAC isoforms were predicted and 64 HDAC-specific peptides with respective four-amino acid long natural flanking sequences were compiled into three QconCAT proteins (S1 Fig, S1 Table, S2 Table and S3 Table). Amino acid sequences were translated into cDNA and cloned into pET21a expression vectors with NdeI/HindIII restrictions sites by Biomatik Corporation (Cambridge, ON, Canada). Expression vector, which included His_6_-tag expressed on C-terminus of the protein, was transformed into One Shot BL21(DE3) *E*. *coli* and cells were cultivated at 37°C in M9 minimal medium containing 1 g/L ^15^NH_4_Cl as the sole nitrogen source. Initial inoculation began with 5 mL LB media and cells were grown for 6 h at 37°C. Cells were pelleted by centrifugation at 20,000 *g* for 20 min and washed with 10 mL of ^15^NH_4_Cl-containing M9 medium. Cells were then transferred to 50 mL ^15^NH_4_Cl-containing M9 medium and grown for 12 h to 14 h at 37°C. Cells were collected by centrifugation at 20,000 *g* for 20 min and washed twice by 100 mL ^15^NH_4_Cl M9 medium. Cells were then transferred to 500 mL ^15^NH_4_Cl M9 medium. Expression was induced with 1 mmol/L IPTG at OD_600_ of 0.6 to 0.8 and incubated for an additional 3 h at 37°C. Cells were divided into 10 portions and harvested by centrifugation at 20,000 *g* for 30 min. One portion of cells was resuspended in 20 mL of lysis buffer (50 mmol/L Tris, pH 7.5). Cells were lysed by sonication and centrifuged at 20,000 *g* for 30 min; supernatant was discarded. Pellet was resuspended in 3 mL urea buffer (7 mol/L urea; 0.1 mol/L NaH_2_PO_4_; 0.01 mol/L Tris•Cl; pH 8.0) and ^15^N-labeled QconCAT was purified on nickel-nitrilotriacetic (Ni-NTA) acid resin (Qiagen, Valencia, CA). Purified ^15^N-labeled QconCAT was loaded onto a SpinTrap G-25 spin column (GE Healthcare, Waukesha, WI) to exchange buffer into 25 mmol/L NH_4_HCO_3_ with 1% (mass fraction) SDS. Protein concentration was measured in presence of 1% (mass fraction) SDS using detergent-compatible DC Protein Assay kit and bovine serum albumin as standard. Final QconCATs were aliquoted and stored at -80°C. Purity was estimated by 10% sodium dodecyl sulfate-polyacrylamide gel electrophoresis (SDS-PAGE) and ImageJ software (http://www.imagej.nih.gov/ij/).

Intact masses of QconCATs were determined experimentally using an Agilent 6550 QTOF and mass deconvolution with MagTran 1.0 software. QconCATs were eluted from an Agilent ProtID C18 nanochip (75 μm x 150 mm, 300 nm) over 10 min gradient from 20% to 80% acetonitrile containing 0.1% (volume fraction) formic acid at a flow rate of 400 nL/min. Acquisition method in positive mode used capillary temperature 275°C, fragmentor 180 V, capillary voltage 1950 V, and a 500 m/z to 2000 m/z mass window. QconCAT characterization on the peptide level was performed using an Applied Biosystems 4700 Proteomic Analyzer (Framingham, MA). For sequence confirmation, 50 pmol of each QconCAT was digested for 15 h at 37°C with trypsin (50:1, mass ratio) in 25 mmol/L NH_4_HCO_3_. Tryptic peptides were mixed with α-cyano-4-hydroxycinnamic acid matrix and analyzed by reflector mode and all expected peptides were observed. To determine level of ^15^N incorporation, peptide spectra were acquired in reflector mode which provided isotopic peaks used to calculate percent ^15^N with Isotopic Enrichment Calculator (www.nist.gov/mml/analytical/organic/isoenrichcalc.cfm). Representative short and long peptides were chosen for each QconCAT to determine percent ^15^N (S2 Fig). HDAC6 full-length protein was used as standard protein for calibration curves and lower limit of quantification (LLOQ) of HDAC6 peptides present in QconCAT#2.

### Human tissues

Authors received frozen samples of frontal cortex from Washington University School of Medicine Alzheimer’s Disease Research Center (St. Louis, MO). Human frontal cortex was collected in accordance with guidance from the Washington University Human Research Protection Office (HRPO number: 89–0556). The authors consulted the Washington University HRPO, which determined that Institutional Review Board (IRB) oversight was not required for this study and waived the need for IRB approval. In the state of Missouri, individuals can give prospective consent for autopsy. Our participants provided this consent by signing the hospital’s autopsy form. If the participant does not provide future consent before death, the DPOA or next of kin provide it after death. Human retina use conformed to the Declaration of Helsinki and had been approved by the Ethical Committee at Case Western Reserve University. Eyes were acquired, characterized and dissected as described [27]. The neural retina was isolated, flash-frozen in liquid nitrogen, and stored at -80°C until analyzed. Handling of tissues for sample processing conformed to University of Maryland regulations. All data were analyzed anonymously. Demographic information on the de-identified donors is summarized in S4 Table.

### Human frontal cortex processing

Minced frontal cortex tissue was homogenized in 25 mmol/L NH_4_HCO_3_ by sonication at 30 W using five 10 s continuous cycles (Sonicator 3000, Misonix Inc., Farmingdale, NY). Homogenates were centrifuged for 5 min at 2,000 *g* to remove debris. Resulting supernatant was measured for total protein concentration using detergent-compatible DC protein assay kit in the presence of 1% (mass fraction) SDS and bovine serum albumin as a standard. Homogenates were stored at -80°C. Pools of 20 mg total protein were prepared from equal amounts of each AD (n = 5) and normal (n = 5) donors. Samples were centrifuged at 106,000 *g* for 1 h at 4°C. Supernatant, which contained soluble protein and HDAC, was supplemented with 10 pmol of each QconCAT, 20 mmol/L DTT, and 1% (mass fraction) SDS. After 1 h incubation at room temperature to reduce cysteines, 55 mmol/L iodoacetamide was added and incubated for an additional 1 h to alkylate cysteines. Protein was isolated by chloroform/methanol precipitation. Protein pellets were sonicated in 1 mL 25 mmol/L NH_4_HCO_3_ and 0.1% (mass fraction) RapiGest SF (Waters, Milford, MA) then treated in 25:1 mass ratio with trypsin overnight at 37°C. Following trypsinolysis, 0.5% trifluoroacetic acid (TFA) was added and incubated at 37°C for 1 h to cleave acid-labile RapiGest. Samples were then centrifuged at 179,000 *g* for 30 min at 4°C to remove precipitated surfactant. Supernatants were dried using a Vacufuge (Eppendorf AG, Hamburg, Germany).

### Human retina tissue processing

Three pools of post-mortem neural retina from human donors were prepared: normal retina from AD-unaffected (n = 4) and affected (n = 3) donors and retina from donors (n = 4) with age-related macular degeneration (AMD). Pools with total protein amounts of 7.6 mg for normal retina, 8.8 mg for AMD, and 12.3 mg for AD were supplemented with 5 pmol to 7 pmol of QconCAT standards. Samples were further processed similar to human frontal cortex. Protein pellets after chloroform/methanol precipitation were sonicated in 400 μL 25 mmol/L NH_4_HCO_3_ and 0.1% RapiGest then treated in 25:1 mass ratio with trypsin overnight at 37°C. After trypsinolysis, 0.5% (volume fraction) TFA was added and incubated at 37°C for 1 h. Samples were then centrifuged at 179,000 *g* for 30 min at 4°C and supernatants were dried using a Vacufuge. Samples were reconstituted in 300 μL 50% acetonitrile and centrifuged again at 65,000 *g* for 1 h at 4°C to remove insoluble material before final drying in Vacufuge.

### Mice

All animal-handling procedures were approved by the Institutional Animal Care and Use Committee at Case Western Reserve University and conformed to the standards of the U.S. Public Health Service Policy on Humane Care and Use of Laboratory Animals and recommendations of the American Veterinary Association Panel on Euthanasia. 5XFAD mice were obtained by crossing 5XFAD hemizygous males on B6SJL background (purchased from the Jackson laboratory) with wild type B6SJL females (also from The Jackson Laboratory). Only F1 males homozygous with respect to the transgene were used. Age-matched males on C57BL/6J background served as controls. Mice were housed in the Animal Resource Center at Case Western Reserve University and maintained in a standard 12 h light/12 h dark cycle environment. Water and food were provided ab libitum. Mice were sacrificed by cervical dislocation at the age of 4 months, and their brains were immediately isolated with the left hemisphere used for quantifications.

### Mouse brain processing

Left hemispheres of the brain from 5XFAD male mice [28] (n = 4) and age- and sex-matched wild type controls (n = 2) were prepared similar to human frontal cortex and total protein was measured. Mouse brains were processed individually with no sample pooling. Five mg total protein of each mouse brain sample was supplemented with 5 pmol of QconCATs. Further sample processing was consistent with that of human brain. BLAST search of human-derived HDAC peptides in QconCATs was performed for mice and peptides present in mice were used in analysis of mouse brain samples.

### LC-MS/MS analysis

Dried peptides were reconstituted in 3% acetonitrile, 97% water, and 0.1% formic acid (volume fraction). Separation and MRM analysis was performed on an Agilent Zorbax Eclipse Plus C18 RRHD column (2.1 mm x 50 mm, 1.8 μm particle) coupled to Agilent 6490 Triple Quadrupole LC/MS system with iFunnel technology (Santa Clara, CA). Peptides were eluted over 30 min gradient from 15% to 35% acetonitrile containing 0.1% (volume fraction) formic acid at a flow rate of 200 μL/min. Acquisition method used following parameters in positive mode: fragmentor 380 V, cell accelerator 4 V, electron multiplier 500 V, and capillary voltage 3500 V. Collision energy was optimized for each peptide using the default equation from Agilent, CE = 0.036 *m/z*—4.8 [29]. Dwell times were varied based on complexity of samples being analyzed and were in 80 ms to 120 ms range.

### Data analysis

MRM transitions were predicted using OrgMassSpecR (http://orgmassspecr.r-forge.r-project.org/) and 2+ charge precursor ions were selected. Transitions were screened in digest of QconCATs to obtain the 3 or 4 most intense transitions, which were further used for quantification. Relative signal ratios of transitions for quantification were similar in both the standard QconCAT digest and when QconCATs were added into tissue homogenates, indicating no obvious interference from biological matrices on the quantification using selected transitions. Ratio of peak areas for unlabeled biological peptides to fully-labeled ^15^N standard peptides was performed using MassHunter (Agilent) and pmol/mg concentrations were calculated based on known picomoles of standard and milligrams of total protein. Protein concentrations represent the mean ± standard deviation (SD) of transitions and peptides associated with each protein. For human frontal cortex and mouse brain, statistical significance of mean differences between disease and control was determined by Student’s *t*-test, performed separately for each HDAC. For human neural retina, one way ANOVA with Tukey HSD post hoc analysis was performed for each HDAC to determine statistical significance of mean differences between control and disease states. For a cross-tissue comparison of each HDAC, an ANOVA and Tukey HSD post hoc analysis was applied to confirm statistical significance. All pairwise comparisons from ANOVA and post hoc tests are provided in S5 Table.

## Results and Discussion

### Design of QconCATs

Tryptic peptides were predicted for all HDAC isoforms. Peptides that contained less than eight amino acids were removed to avoid small m/z values. Long peptides were excluded because they tend to have lower signal intensities and more charge states with electrospray ionization. In addition to size constraints, peptides containing methionine were avoided due to oxidation and cysteines were avoided due to potential disulfide bonds and oxidation, which would interfere with quantification. Finally, sequences were screened with BLAST to ensure Q-peptides were unique in the human proteome. Q-peptides and their natural flanking sequences were assembled into QconCATs. The purpose of including natural flanking sequences is to reduce disparity in efficiency of tryptic digestion between endogenous protein and QconCAT.[21] Additionally, a His_6_-tag was included on the C-terminal end, which ensured QconCATs purified by Ni-NTA column would be full-length and not include truncated forms. An overview of design and expression is shown in S1 Fig and QconCAT sequences are presented in S1 Table, S2 Table and S3 Table.

### Characterization of ^15^N QconCAT internal standard

**Fig 1:**
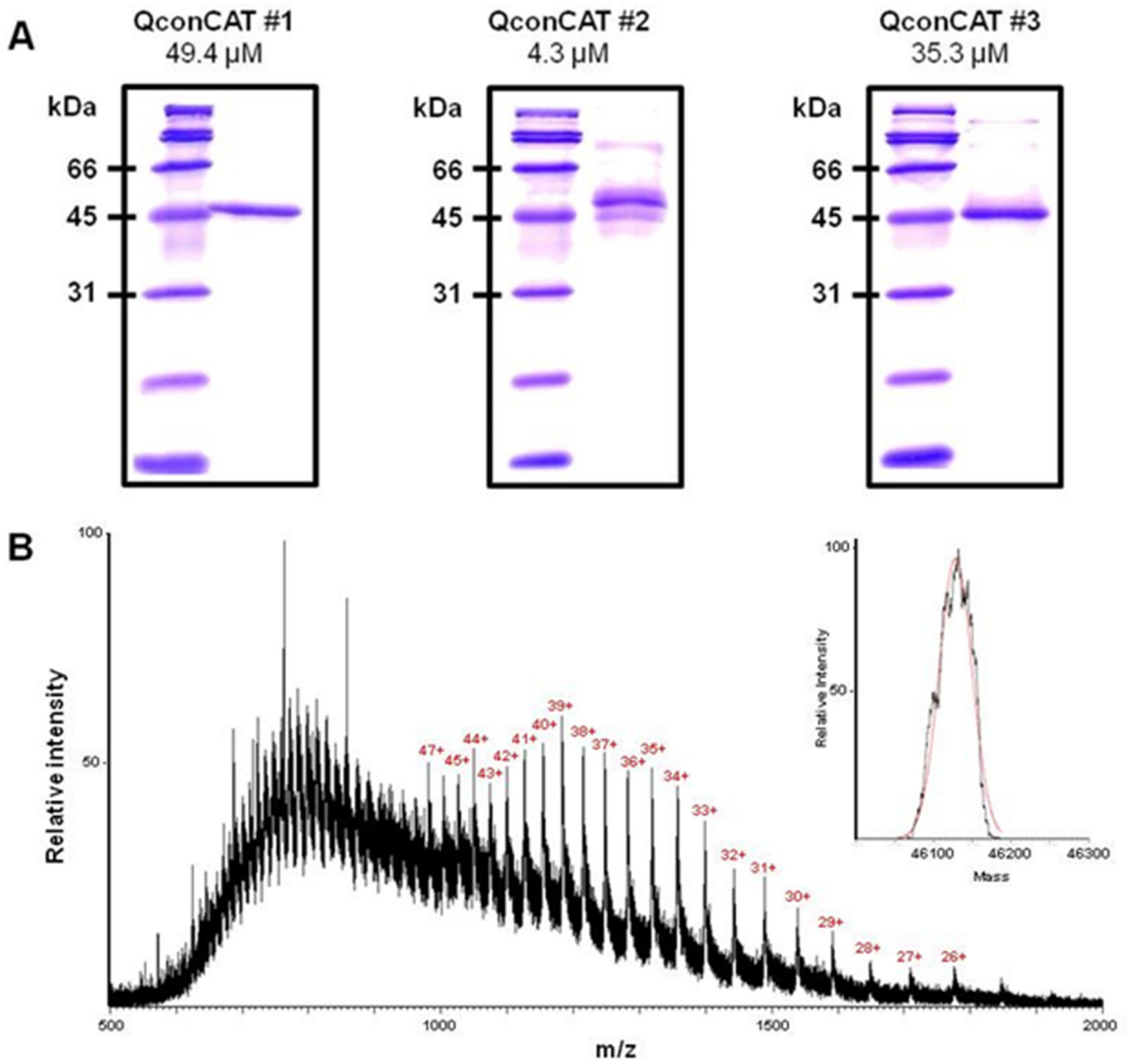
Purity and size of QconCATs. (**A**) Purified QconCATs (30 pmol of QconCAT#1, 22 pmol of QconCAT#2, and 25 pmol of QconCAT#3) were separated on 10% SDS-PAGE and ImageJ software was used for estimation of purity. Purity was nearly 100% for QconCAT#1, ≥95% for QconCAT#2, and ≥97% for QconCAT#3. Protein concentrations were determined by DC Protein Assay. (**B**) Charge state masses were collected for all QconCATs (representative spectrum of QconCAT#1 shown) with an Agilent 6550 QTOF and deconvoluted with MagTran 1.0 (insert) to confirm protein mass was congruous with expected mass based on sequence. Observed mass of QconCAT#1 was 46126.7 Da (46125.6 Da theoretical), QconCAT#2 was 48931.2 Da (48929.7 Da theoretical), and QconCAT#3 was 43167.8 Da (43166.6 Da theoretical).

**Fig 2:**
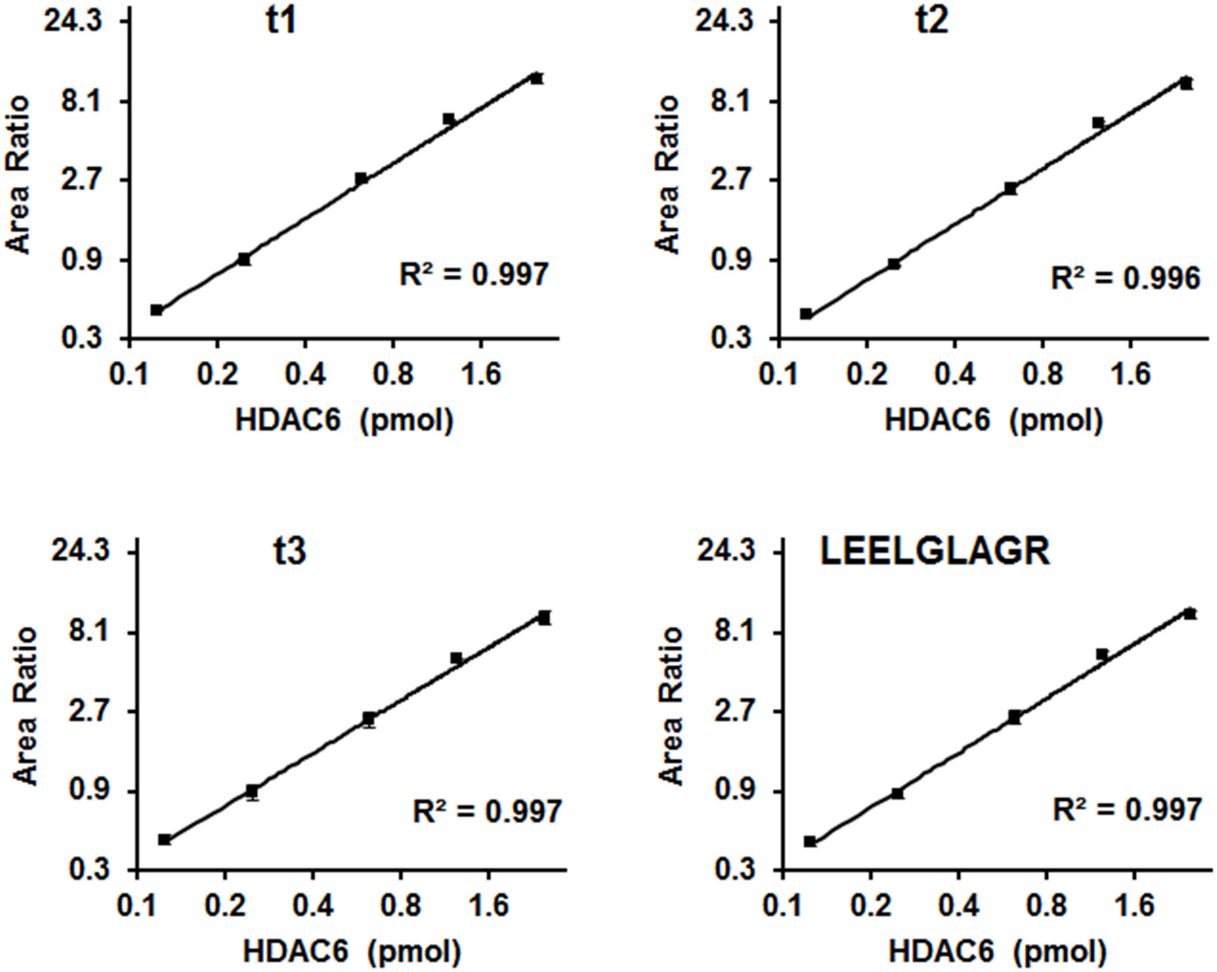
Calibration curves for quantification of peptide LEELGLAGR from HDAC6 standard using ^15^N-labeled peptide from QconCAT#2 as internal standard. Area ratio of ^14^N-LEELGLAGR to ^15^N-LEELGLAGR for each of the three transitions for quantification was plotted versus pmol of HDAC6 standard. Individual transitions shown are (**t1**) 479.3/586.4 and 485.3/595.3, (**t2**) 479.3/715.4 and 485.3/725.4, (**t3**) 479.3/586.4 and 485.3/595.3. Three replicates for each were collected and presented as mean ± SD. Consensus for t1–t3 is represented in LEELGLAGR graph.

Purity of QconCATs was estimated by SDS-PAGE (Fig 1A) and ImageJ software to be nearly 100% for QconCAT#1, ≥95% for QconCAT#2, and ≥97% for QconCAT#3. No correction for protein concentration was made for analysis. Intact mass of each QconCAT was consistent with expected mass, indicating full-length expression of protein. Fig 1B shows a representative spectrum and deconvolution for QconCAT#1. Deconvoluted mass for QconCAT#1 was 46126.7 (46125.6 Da theoretical), QconCAT#2 was 48931.2 Da (48929.7 Da theoretical), and QconCAT#3 was 43167.8 Da (43166.6 Da theoretical).^15^N isotope incorporation (S2 Fig) was determined using a relatively short and long peptide for each QconCAT and both peptide lengths yielded consistent results. Incorporation was 99.3% for QconCAT#1, 99.3% for QconCAT#2, and 99.4% for QconCAT#3. These values were accepted as complete labeling and no correction was applied to data. Calibration curves were performed using variable amounts of unlabeled HDAC6 mixed with a fixed amount of QconCAT#2, which contained HDAC6 peptides. Calibration curves showed linearity of Q-peptides in the 0.125 pmol to 2.5 pmol HDAC6 range tested; Fig 2 shows a representative calibration curve for peptide LEELGLAGR. Lower limit of quantification (LLOQ) was defined as the lowest point of calibration curve that could be measured with a coefficient of variance less than 20%. LLOQ for HDAC6 peptide LEELGLAGR was 0.05 pmol in mixture of HDAC6 standard and QconCAT#2 internal standard.

### Human frontal cortex

The outermost portion, or cortex, of the frontal lobe was used for sample preparation. Cortex is commonly referred to as gray matter and consists primarily of neural cell bodies with few myelinated axons. Measurements in frontal cortex present a challenge due to the high lipid content, approximately 40% of dry weight [30]; therefore sample processing is important. Two methods of fractionation were used to reduce sample complexity while maintaining the same ratio of analyte to internal standard. High speed centrifugation at 106,000 *g* was performed which separated membrane associated proteins and some lipids from soluble proteins and HDACs. MRM measurements performed in the pellet fraction confirmed HDACs were absent in the pellet and only present in the supernatant from high speed centrifugation. Additionally, chloroform/methanol precipitation was performed which removed SDS, by-products of cysteine alkylation, remaining lipids, and salts. These two fractionation steps are important for the success of the mass spectrometry method of quantification, improving chromatographic performance and reducing ion suppression effects.

Measurements were performed on pooled samples from AD (n = 5) and normal (n = 5) human frontal cortex. The purpose of using pooled tissue samples is to minimize the effects of donor-to-donor variation while maintaining the biological variation between donor groups, allowing for evaluation of substantive characteristics of the donor population. This research functions as a feasibility study, demonstrating the applicability of QconCAT technology with MRM MS to conduct protein quantification in a complex biological matrix, such as brain. While our study findings are based on pools from five donors in each sample group, our method can easily be applied to a large number of individual donor samples, if a larger number of samples are available. HDAC isoforms detected in human frontal cortex are summarized in Table 1. Out of the 11 isoforms, we detected HDAC1,2, HDAC5, and HDAC6 only. Other HDAC isoforms are either (i) not significantly expressed in human frontal cortex or (ii) expressed at levels in human frontal cortex that are below the limit of quantification of our MRM assay, which is approximately 5 fmol/mg total protein. Concentration of HDAC1,2 decreased 32% from 1.10 pmol/mg in control to 0.746 pmol/mg in AD. HDAC1,2 measurement was based on a peptide in both isoforms because HDAC1 and HDAC2 have similar sequence homology and unique peptides included in QconCATs did not produce signals sufficient for detection or quantification. Therefore, we were not able to ascertain whether both HDAC1 and HDAC2 decreased but were able to determine the sum of their concentrations decreased in AD. HDAC5 and HDAC6 both showed an increasing trend of 47% and 31%, respectively. Work by others has shown that HDAC5 is correlated to memory loss [13] and HDAC6 is correlated with protein aggregation [9,31,32] in mouse models.

**Table 1:** Summary of human frontal cortex HDAC concentrations.

	pmol/mg tissue protein^a^	
Isoform	Control	AD
HDAC1,2	1.10 ± 0.14	0.746 ± 0.071^c^
HDAC5	0.083 ± 0.016	0.122 ± 0.025^b^
HDAC6	0.106 ± 0.015	0.139 ± 0.015^c^

^a^Measurements were performed on supernatant of frontal cortex from age-matched control (n = 5) and severe AD (n = 5) human donor pools (S4 Table) supplemented with QconCAT standard after high-speed centrifugation. Concentrations were calculated for three biological replicates and with three transitions per Q-peptide. Q-peptide transitions are summarized in S6 Table. Data presented as mean ± SD.

^b^, p < 0.01;

^c^, p < 0.001.

Immunohistochemical staining of cerebral cortex in The Human Protein Atlas (www.proteinatlas.org) shows HDAC2 is highly abundant, and when combined with less expressed HDAC1, they would be among the most abundant. They report HDAC5 is present at medium levels and HDAC6 at low levels. While our concentrations of HDAC5 and HDAC6 are within a similar range and in a specific portion of the cerebral cortex, called the frontal cortex, the qualitative levels on these HDACs available in The Human Protein Atlas are similar to our profile. Additionally, quantitative polymerase chain reaction assays by Jakovceoski et al. have shown transcript levels of HDAC1, 2, 5, and 6 are nearly identical in frontal cortex [33], which is consistent with our findings given that HDAC1 and 2 are combined. HDAC6 has been shown to increase in AD brain [34], as we have shown. However, it has been reported that HDAC2 increases and HDAC1 remains normal in AD brain and cortex [35,36], counter to our observed trends.

### Human retina

Neural retina is a specialized extension of the central nervous system (CNS) connected to the occipital lobe of the brain. We selected neural retina to test our protocol of HDAC quantification in a different biological matrix and to gain insight into AD changes in other functionalized portions of the CNS. Sample processing procedures for frontal cortex were also applied to retina, enabling quantification of HDACs in this tissue (Table 2). Similarity in HDAC coverage acquired in frontal cortex demonstrates this procedure is also appropriate for neural retinal tissue matrix. Retina samples were obtained from AD-affected and AMD donors to evaluate changes in HDACs in these disease states. AMD retina was included because it is another degenerative disease associated with aging and has several clinical similarities with AD, including β-amyloid deposition and stress stimuli [25,37,38]. HDAC concentrations in retina are summarized in Table 2. A decrease in concentration was observed for all detected HDAC isoforms in normal retina from AD-affected donors compared to normal retina from AD-unaffected donors. HDAC1,2 decreased similarly in both AD-affected tissue types of retina and frontal cortex and showed a slight decrease in AMD retina. HDAC5 and HDAC6 showed a decrease in AMD retina and a greater decrease in retina from AD-affected donors, counter to the increasing trend observed in frontal cortex. HDAC7 did not appear to change in concentration in AMD retina but was nearly reduced in half in retina from AD-affected donors. In addition to trends in concentration of retinal HDACs and their comparison to those in frontal cortex in disease state, overall profile of HDAC isoforms was also different in retina and frontal cortex. HDAC1,2 and HDAC5 had similar levels of concentration in both frontal cortex and retina tissues while HDAC6 in retina was approximately five-fold greater than the concentration in control frontal cortex. Additionally, HDAC 7 was detected in retina in concentrations comparable to HDAC5 in retina but was not detected in frontal cortex. Disparity in changes in retina from those in frontal cortex is an interesting pathological phenomenon that may suggest importance of HDAC isoforms and their respective roles in specialized tissues. HDAC1, 3, and 6 have been shown by Western blot to decrease in concentration in retina as it ages, while HDAC2 and 5 remain unchanged [39]. We also observed a decrease in HDAC1,2 and in HDAC6 in disease-affected retina, perhaps indicative of their decreased abundance in aging tissue.

**Table 2:** HDAC concentrations in human neural retina.

	pmol/mg tissue protein^a^		
Isoform	Control	AD	AMD
HDAC1,2	1.63 ± 0.12	0.743 ± 0.065^b^	1.14 ± 0.19^b^
HDAC5	0.106 ± 0.014	0.0393 ± 0.0077^b^	0.074 ± 0.017^b^
HDAC6	0.558 ± 0.081	0.298 ± 0.062^b^	0.415 ± 0.032^b^
HDAC7	0.078 ± 0.020	0.0251 ± 0.0068^b^	0.069 ± 0.017

^a^Measurements were performed on supernatant of retina from control (n = 4), AD-affected (n = 3), and AMD (n = 4) human donor pools (S4 Table) supplemented with QconCAT standards after high-speed centrifugation. Concentrations were calculated for three experimental replicates and with three transitions per Q-peptide. Q-peptide transitions are summarized in S6 Table. Data presented as mean ± SD.

^b^, p < 0.01.

### Mouse brain

Whole hemisphere of brain was analyzed for several reasons. Firstly, whole hemisphere of mouse brain was homogenized during sample preparation and HDAC concentrations reflect inclusive averages for the entire brain rather than a specific, functionalized portion of human brain, such as frontal cortex. Secondly, tissue matrix varies from human frontal cortex, which mainly contains only gray matter, to the brain hemisphere of mice, which includes white matter rich in myelinated axons. Lipids are a concern in brain tissue analysis and whole brain tissue contains more lipids than gray matter of frontal cortex due to the abundance of myelin, which is 80% lipid by dry weight [30]. Thirdly, and perhaps most important for AD pathology, HDAC profiles in mouse models may be compared to that of human frontal cortex. There are two main points to consider in comparing mouse and human HDAC profiles for AD: species variation and familial versus sporadic AD. While the eleven main isoforms of HDACs are present in both mice and humans, there are some differences in the amino acid sequences that had to be taken into account. Accordingly for mouse sample analysis, BLAST was used to refine human peptides present in expressed QconCATs to those for both human and mouse isoforms, approximately half of total human peptides. Thus, not all peptides measured in human were used to obtain HDAC concentrations in mice. Additionally, HDAC isoform concentrations could be dictated by levels of HDAC regulation in mice different from that in humans. Another important caveat to using animal models for AD research is that mice may only be produced as models of familial AD via known mutations. The vast majority of AD cases in humans are considered sporadic AD and cannot be predicted. While clinical indicators of AD, such as amyloid aggregation, may be present in both sporadic and familial AD, pathogenesis may vary. In this study, 5XFAD transgenic mouse models of rapid brain amyloidosis were used. 5XFAD mice overexpress two mutant human proteins: presenilin 1 with familial AD (FAD) mutations M146L and L286V and β-amyloid precursor protein (APP695) with Swedish (K670N, M671L), Florida (I716V), and London (V717I) FAD mutations. Mixed wild type B6SJLF1/J mice are used for the production of 5XFAD transgenic mice and therefore provide an appropriate background strain of wild-type mice, which were used as our control. HDAC isoform concentrations in both control and 5XFAD mice are summarized in Table 3. HDAC1,2 had no change in 5XFAD mice but decreased by 1.5-fold in AD human frontal cortex (Table 1). Since HDAC1,2 measurements were based on a conserved peptide, we were unable to determine the contribution of isoforms HDAC1 and HDAC2 to the reported measurements. HDAC3 had a slight increase of 50%, HDAC4 also increased by 63%, and HDAC5 and HDAC6 did not show significant change in 5XFAD mouse model. Of the HDAC isoforms reported for both mice and human profiles, HDAC1,2 was most abundant in both. However, HDAC3 and HDAC4 were within the concentration range of other isoforms in HDAC profile in mouse model yet not detected in human. Inconsistency in observed HDAC profiles of human and mouse brains may be for several reasons, such as differences in expression of HDACs between species, differences in functionalization of frontal cortex to whole brain hemisphere, and post-translational modification of peptides used for quantification. Additionally, due to sampling limitations, small portions of frontal cortex tissue were excised from human donors at random by support pathologists. It may be possible that patterning of HDACs varies across the frontal cortex region of the brain. While our pilot study highlights specific HDAC changes, future studies using our MRM assay could be performed on many sites across a particular donor to address variability in HDAC concentrations within frontal cortex. Mouse models allow for knockout and overexpression studies for disease pathology but our work suggests HDAC findings in mouse models should not be over interpreted without evaluation of human tissue.

**Table 3:** HDAC concentrations in whole hemisphere of mouse brain.

	pmol/mg tissue protein^a^	
Isoform	WT	5XFAD
HDAC1,2	1.010 ± 0.074	1.02 ± 0.23
HDAC3	0.131 ± 0.010	0.196 ± 0.024^b^
HDAC4	0.230 ± 0.029	0.375 ± 0.023^c^
HDAC5	0.154 ± 0.015	0.143 ± 0.023
HDAC6	0.0149 ± 0.0039	0.0171 ± 0.0022

^a^Measurements were performed on supernatants of whole left hemispheres of mouse brains from 5XFAD (n = 4) and wild type (n = 2) mice supplemented with QconCAT standards after high-speed centrifugation. Three transitions per Q-peptide were used to calculate concentration for each mouse. Wild type (n = 2) and 5XFAD (n = 4) mice concentrations were averaged and data is presented as mean ± SD of each sample group. Q-peptide transitions are summarized in S6 Table.

^b^, p < 0.05;

^c^, p < 0.01.

HDAC profiles in mouse brain were evaluated by Broide et al. [18] using *in situ* hybridization and they found that HDAC3, 4, and 5 were close in abundance with HDAC6 being less abundant and the sum of HDAC1 and 2 being more abundant, consistent with our observations. Wang et al. performed autoradiography with a radioactive isotopologue to an inhibitor of HDAC1-3 and 8 to measure pmol/mg concentrations *ex vivo* in mouse brain [40]. They were unable to detect HDAC8, as we were unable to, which was confirmed by Western blot. They reported the sum of HDAC1-3 concentrations to be 12.9 pmol/mg within mouse brain, more than our measurement of 1.14 pmol/mg. While their method was able to provide pmol/mg concentrations, this was based on binding to an inhibitor shared between multiple HDACs and individual HDAC measurements would have to be derived by using Western blot intensities of HDAC1, 2, and 3 performed in parallel to account for contribution of each HDAC isoform bound to the labeled inhibitor.

Measurements for HDAC5 and 6 in human tissues and HDAC4 in mouse tissue were performed using two peptides for each isoform (S6 Table). When measured individually, peptides were in close agreement with the other respective peptide for the isoform (S7 Table). This demonstrates that the peptide-to-peptide variability in measurement was not statistically significant using the MRM assay with QconCATs as internal standards.

## Conclusions

Herein, we obtained concentrations of several HDAC isoforms in three different tissues: human frontal cortex, human retina, and whole mouse brain. We demonstrated that our method of protein quantification is suitable in different types of tissues, particularly in challenging matrices with high lipid content. In human frontal cortex, observed HDAC isoforms had different changes in control to AD with HDAC1,2 decreasing, HDAC5 increasing, and HDAC6 having negligible change in concentration. In human retina, reduction in concentration of detected HDACs was observed in both AMD retina and retina from AD donors in comparison to control retina. Mouse brain depicted a slightly different HDAC profile from humans with variance in overall HDAC3 and HDAC4 abundance being greater in mouse. Additionally, HDAC1,2 concentrations in AD-affected donors decreased compared to control in both human frontal cortex and retina but showed no change in mouse hemisphere. Mouse models are widely used for neurodegenerative research and our data indicates some disparity in HDAC profiles and changes in disease state compared to human frontal cortex tissue.

However, limitations in Q-peptides suitable for MRM did restrict our HDAC profile in tissues examined. Many HDAC isoforms have similar sequences and therefore selecting unique sequences that meet MRM criteria, such as length and no post-translational modifications, restricts availability of Q-peptides. Furthermore, some peptides do not perform well either in ionization efficiency or reproducibility of transitions. Once unsuitable peptides are excluded, HDAC isoform profiles were further restricted to isoforms that can be observed in biological sample. The abundance of some HDACs may be present at very low levels precluding their detection via MRM and may require more sensitive approaches, such as an enzyme-linked immunosorbent assay (ELISA). While the QconCAT MRM method may have shortcomings, it is able to multiplex protein quantification, reduce analysis time, and accounts for loss during sample processing compared to synthetic labeled peptides and ELISA [20].

Our feasibility study demonstrates the value of MRM mass spectrometry and QconCAT technology to perform multiplexed, quantitative measurements of HDAC isoforms in various tissues. Moreover, our measurements show that changes in concentrations of HDACs have different trends in frontal cortex and neural retina in AD and that mouse hemisphere and human frontal cortex HDAC profiles differ. This method may be applied to other tissues and disease conditions to obtain concentrations of HDAC isoforms, a practical and useful tool for investigation of disease pathology.

## Supporting Information

S1 Fig QconCAT design overview. (DOCX)

S2 Fig ^15^N incorporation in QconCATs. (DOCX)

S3 Fig Representative chromatogram and spectrum. (DOCX)

S1 Table HDAC QconCAT#1 sequence and peptides for quantification. (DOCX)

S2 Table HDAC QconCAT#2 sequence and peptides for quantification. (DOCX)

S3 Table HDAC QconCAT#3 sequence and peptides for quantification. (DOCX)

S4 Table Donor information. (DOCX)

S5 Table Results of one way ANOVA and Tukey post hoc analysis. (DOCX)

S6 Table Transitions used for quantification. (DOCX)

S7 Table Comparison of individual Q-peptide and protein measurements. (DOCX)
