# Supplementary material for: Quantification of Histone Deacetylase Isoforms in Human Frontal Cortex, Human Retina, and Mouse Brain
Source: PLoS One. 2015 May 11;10(5):e0126592. doi: 10.1371/journal.pone.0126592 (PMC4427357; doi:10.1371/journal.pone.0126592)
Supplement: S1 Fig — (DOCX) [file pone.0126592.s001.docx]

**S1 Fig. QconCAT Design Overview.** Tryptic peptides from HDACs were predicted and screened for peptides suitable for MRM quantification. Natural flanking sequences consisting of four amino acids on each side of tryptic Q-peptides were included. A cDNA construct encoding the concatamer of these peptides was inserted into pET21a expression vector by Biomatik. BL21 (DE3) cells were transformed with plasmid and QconCATs were expressed with His_6_-tag and purified by nickel-nitrilotriacetic (Ni-NTA) acid resin.
