# Supplementary material for: Quantification of Histone Deacetylase Isoforms in Human Frontal Cortex, Human Retina, and Mouse Brain
Source: PLoS One. 2015 May 11;10(5):e0126592. doi: 10.1371/journal.pone.0126592 (PMC4427357; doi:10.1371/journal.pone.0126592)
Supplement: S2 Fig — (DOCX) [file pone.0126592.s002.docx]

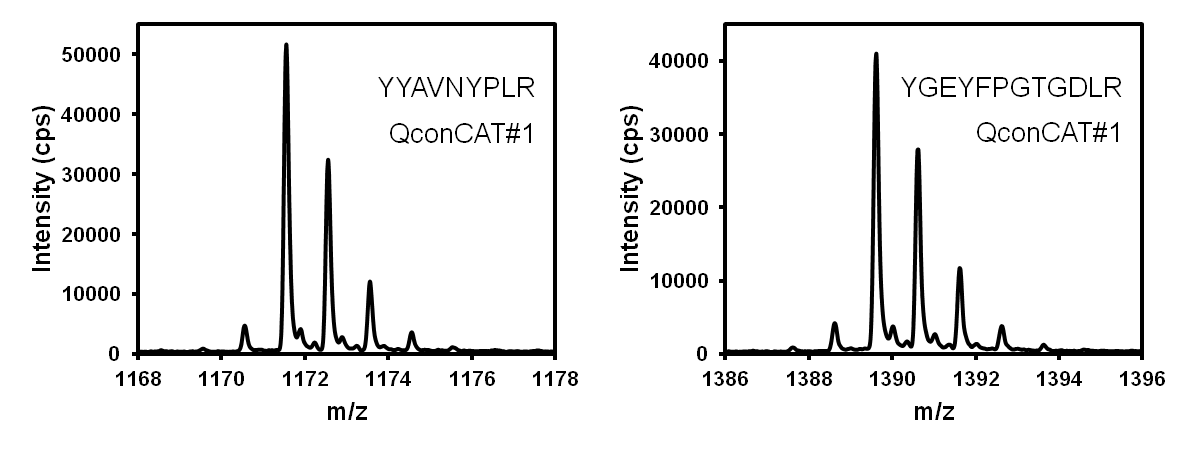


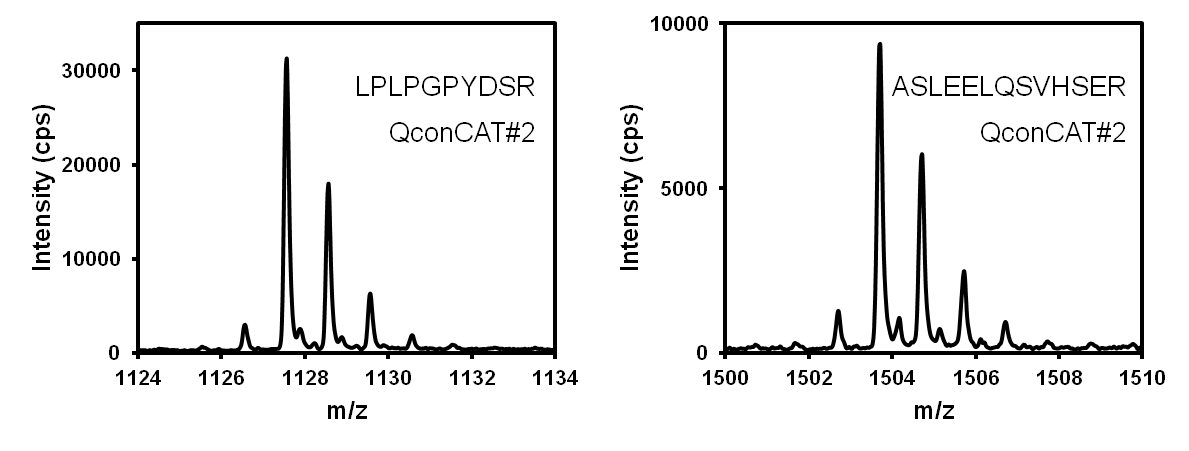

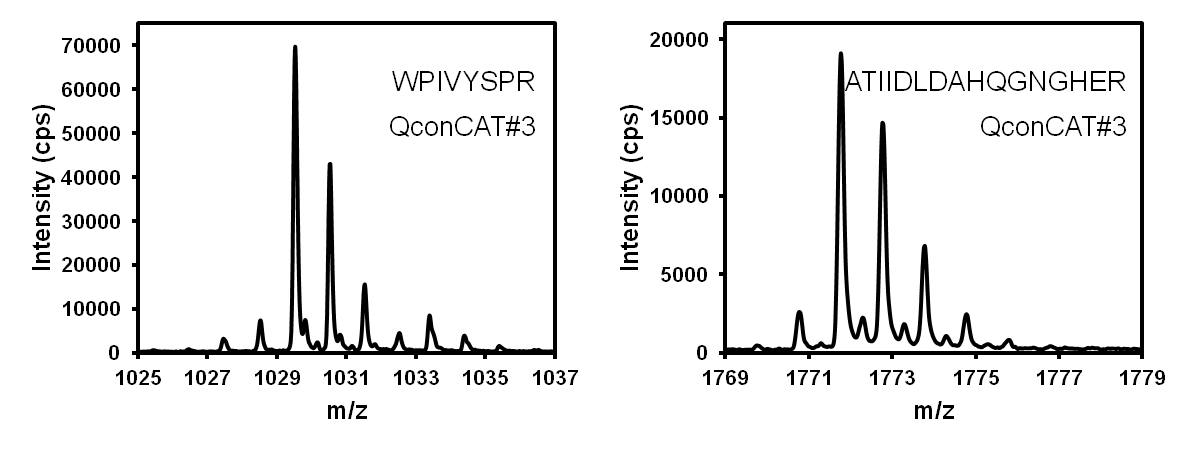


**S2 Fig. ^15^N incorporation in QconCATs.** MALDI spectra of QconCAT peptides were used to calculate ^15^N incorporation with Isotopic Enrichment Calculator (www.nist.gov/mml/analytical/organic/isoenrichcalc.cfm). Incorporation of ^15^N was determined to be 99.3% for QconCAT#1, 99.3% for QconCAT#2, and 99.4% for QconCAT#3.
