# Supplementary material for: Quantification of Histone Deacetylase Isoforms in Human Frontal Cortex, Human Retina, and Mouse Brain
Source: PLoS One. 2015 May 11;10(5):e0126592. doi: 10.1371/journal.pone.0126592 (PMC4427357; doi:10.1371/journal.pone.0126592)
Supplement: S3 Fig — (DOCX) [file pone.0126592.s003.docx]

**
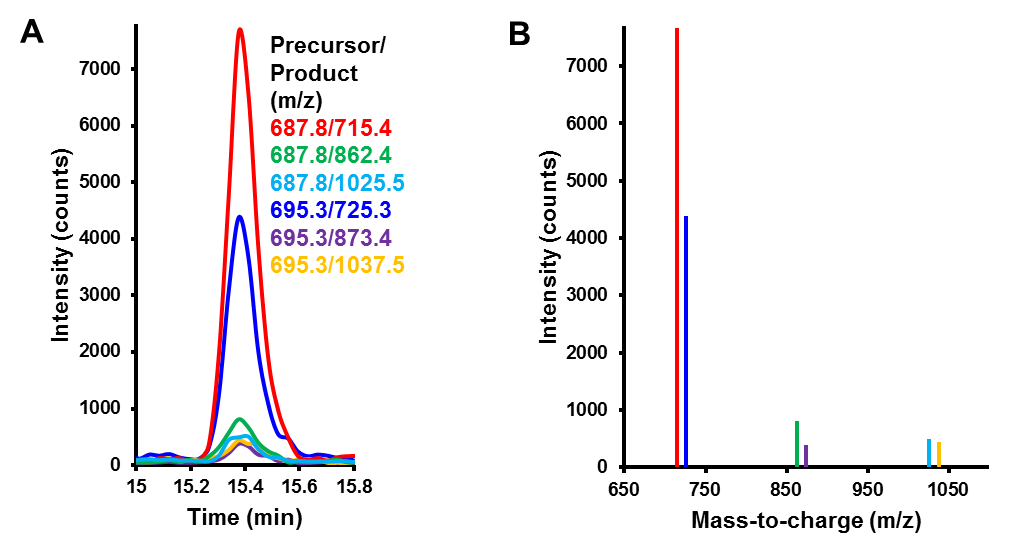
**

**S3 Fig. Representative chromatogram and spectrum.** Transitions for HDAC1,2 peptide YGEYFPGTGDLR in 5XFAD mouse brain hemisphere are shown as a (A) chromatogram and (B) spectrum. Chromatogram peaks of transitions are representative of data for other peptides in neural tissue. Peak areas were used for quantification.
