# Supplementary material for: Quantification of Histone Deacetylase Isoforms in Human Frontal Cortex, Human Retina, and Mouse Brain
Source: PLoS One. 2015 May 11;10(5):e0126592. doi: 10.1371/journal.pone.0126592 (PMC4427357; doi:10.1371/journal.pone.0126592)
Supplement: S1 Table — (DOCX) [file pone.0126592.s004.docx]

**S1 Table. HDAC QconCAT#1 sequence and peptides for quantification.**

**HDAC QconCAT #1**

MEMTKYHSDDYIKFLRSGKGKYYAVNYPLRDGIDSFHKYGEYFPGTGDLRDIGAEMTKYHSDEYIKFLRSDMCRFHSEDYIDFLQRVSPTFCSRYTGASLQGATQLNNKICDIVPLRDGIDDQSYKHLFQTYDRTDEADAEERGPEELSGRDQPVELLNPARVNHMMDLRLDHQFSLPVAEPALREQQLLAMKHQQELLEHQRKLEREKGKESAVASTEVKMKLQQMNKIIPKPSEPARQPESEELREHQALLDEPYLDRLPGQPGQKEAHAQAGVQVKKQEPPGQRQPSEQELLFRQQALGMCKVAINWSGGWHHAKKDEAGKGRYYSVNVPIQDGIQDEKYYQISVLKEVYQAFNPKAVVLERHRIQQILNYIKGNLKKLAAALEHHHHHH

**Sequence Isoforms**

EMTKYHSDDYIKFLRS HDAC1

GKGKYYAVNYPLRDGID HDAC1

SFHKYGEYFPGTGDLRDIGA HDAC1, 2

EMTKYHSDEYIKFLRS HDAC2

DMCRFHSEDYIDFLQRVSPT HDAC3(all)

FCSRYTGASLQGATQLNNKICDI HDAC3(all)

VPLRDGIDDQSYKHLFQ HDAC3(all)

TYDRTDEADAEERGPEE HDAC3(all)

LSGRDQPVELLNPARVNHM HDAC4

MDLRLDHQFSLPVAEPALREQQL HDAC4

LAMKHQQELLEHQRKLER HDAC4

EKGKESAVASTEVKMKLQ HDAC4

QMNKIIPKPSEPARQPES HDAC4

EELREHQALLDEPYLDRLPGQ HDAC4

PGQKEAHAQAGVQVKKQEP HDAC4

PGQRQPSEQELLFRQQAL HDAC4

GMCKVAINWSGGWHHAKKDEA HDAC8(1-3, 5)

GKGRYYSVNVPIQDGIQDEKYYQI HDAC8(1-2, 4-5)

SVLKEVYQAFNPKAVVL HDAC8(1, 4)

ERHRIQQILNYIKGNLK HDAC8(1, 4)

Molecular weight: 45547.3 Da ^14^N (46125.6 Da ^15^N)

Grand average of hydropathicity (GRAVY): -0.885 (hydrophilic)
