# Supplementary material for: Quantification of Histone Deacetylase Isoforms in Human Frontal Cortex, Human Retina, and Mouse Brain
Source: PLoS One. 2015 May 11;10(5):e0126592. doi: 10.1371/journal.pone.0126592 (PMC4427357; doi:10.1371/journal.pone.0126592)
Supplement: S2 Table — (DOCX) [file pone.0126592.s005.docx]

**S2 Table. HDAC QconCAT#2 sequence and peptides for quantification.**

**HDAC QconCAT #2**

MSGREPSLEILPRTSLHVELRGALVGSVDPTLREQQLHLTRQHEVQLQKHLKQPSYKLPLPGPYDSRDDFPASPKLSTQQEAERQALQGGMKSPPDQPVKHLFTIWSRLQETGLLSKCERIDSKKLLGPISQKMYAVHAIKEQLIQEGLLDRCVSFSVLRLVDAVLGAEIRNGMAAGGKLILSLEGGYNLRALAEIMCRLEELGLAGRCLTLVAARHAQTISGHALRILIVALERTVHPNSPGIPYRTLEPIPYRTLEPLETEGATRSMLSPLRKTVSEPNLKLRYKTTERLSGSGLHWPLSRTRSELEHRELGHGQPEARGPAPRGRKASLEELQSVHSERHVLLHSERHVLLYGTNPLSRLKLDDNGKLAGLLAQRMFVMNAARWAAGSVTDLAFKVASRLGNRVDPLSEEGWKQKPNKLAAALEHHHHHH

**Sequence Isoforms**

MSGREPSLEILPRTSLH HDAC5(all)

VELRGALVGSVDPTLREQQL HDAC5(all)

HLTRQHEVQLQKHLKQ HDAC5(all)

PSYKLPLPGPYDSRDDFP HDAC5(all)

ASPKLSTQQEAERQALQ HDAC5(all)

GGMKSPPDQPVKHLFT HDAC5(all)

IWSRLQETGLLSKCERI HDAC5(1, 3)

DSKKLLGPISQKMYAV HDAC5(1, 3)

HAIKEQLIQEGLLDRCVSF HDAC6

SVLRLVDAVLGAEIRNGMA HDAC6

AGGKLILSLEGGYNLRALAE HDAC6

IMCRLEELGLAGRCLTL HDAC6

VAARHAQTISGHALRILIV HDAC6

ALERTVHPNSPGIPYRTLEP HDAC7(1, 3-8)

IPYRTLEPLETEGATRSMLS HDAC7(1, 3-8)

PLRKTVSEPNLKLRYK HDAC7(1, 3-8)

TTERLSGSGLHWPLSRTRSE HDAC7(1, 3-8, 10)

LEHRELGHGQPEARGPAP HDAC7(1, 3-8, 10)

RGRKASLEELQSVHSERHVLL HDAC7(all)

HSERHVLLYGTNPLSRLKLD HDAC7(all)

DNGKLAGLLAQRMFVM HDAC7(all)

NAARWAAGSVTDLAFKVASR HDAC7(all)

LGNRVDPLSEEGWKQKPN HDAC7(all)

Molecular weight: 48288.6 Da ^14^N (48929.7 Da ^15^N)

Grand average of hydropathicity (GRAVY): -0.435 (hydrophilic)
