# Supplementary material for: Quantification of Histone Deacetylase Isoforms in Human Frontal Cortex, Human Retina, and Mouse Brain
Source: PLoS One. 2015 May 11;10(5):e0126592. doi: 10.1371/journal.pone.0126592 (PMC4427357; doi:10.1371/journal.pone.0126592)
Supplement: S3 Table — (DOCX) [file pone.0126592.s006.docx]

**S3 Table. HDAC QconCAT#3 sequence and peptides for quantification.**

**HDAC QconCAT #3**

MQIQKQLLIAEFQKQHENNLTRQHQAQLQEHIKELLAGRERAVASTEVKQKLQPSYKYTLPGAQDAKDDFPQEDRAPSSGNSTRSDSSIWSRLQETGLLNKCERILDPRILLGDDSQKFFSSVIGKDLAPGFVIKVIIGCCRIFPDGVAGREQLLVAGREQLLAQQRMHSMPFLRESDADAVGRGQGLQSARAAQAPHWKSLQQSLAREEALTALGKLLYLAVRRGLSHGAQRLLCVMYLRGQLEPQWKMLQCPASRDPGPGAEWRGTSPETRWPIVYSPRYNITGLEKLHPFDAGKWGKVVEAREASEEDLLVVHTRRYLNGISRATIIDLDAHQGNGHERDFMDEGDRLGGLSISPAGIVKRDELKLAAALEHHHHHH

**Sequence Isoforms**

QIQKQLLIAEFQKQHEN HDAC9(all)

NLTRQHQAQLQEHIKELLA HDAC9(all)

GRERAVASTEVKQKLQ HDAC9(all)

PSYKYTLPGAQDAKDDFP HDAC9(all)

QEDRAPSSGNSTRSDSS HDAC9(1, 3-8)

IWSRLQETGLLNKCERI HDAC9(1-2, 4-7)

LDPRILLGDDSQKFFSS HDAC9(1-2, 4-7)

VIGKDLAPGFVIKVII HDAC9(3, 8)

GCCRIFPDGVAGREQLL HDAC9(8)

VAGREQLLAQQRMHSM HDAC9(8)

PFLRESDADAVGRGQGL HDAC10(all)

QSARAAQAPHWKSLQQ HDAC10(all)

SLAREEALTALGKLLYL HDAC10(1-2, 4)

AVRRGLSHGAQRLLCV HDAC10(1-2, 4)

MYLRGQLEPQWKMLQC HDAC10(1-2)

PASRDPGPGAEWRGTS HDAC10(4)

PETRWPIVYSPRYNIT HDAC11(1)

GLEKLHPFDAGKWGKV HDAC11(all)

VEAREASEEDLLVVHTRRYLN HDAC11(all)

GISRATIIDLDAHQGNGHERDFMD HDAC11(all)

EGDRLGGLSISPAGIVKRDEL HDAC11(all)

Molecular weight: 42602.3 Da ^14^N (43166.6 Da ^15^N)

Grand average of hydropathicity (GRAVY): -0.551 (hydrophilic)
