# Supplementary material for: Quantification of Histone Deacetylase Isoforms in Human Frontal Cortex, Human Retina, and Mouse Brain
Source: PLoS One. 2015 May 11;10(5):e0126592. doi: 10.1371/journal.pone.0126592 (PMC4427357; doi:10.1371/journal.pone.0126592)
Supplement: S4 Table — (DOCX) [file pone.0126592.s007.docx]

**S4 Table. Donor information.**

| **Donor ID** | **Age (y)** | **Gender** | **Frontal cortex and retina status** |
| --- | --- | --- | --- |
| 1 | 90 | F | frontal cortex, normal |
| 2 | 91.3 | M | frontal cortex, normal |
| 3 | 87 | M | frontal cortex, normal |
| 8 | 91.5 | M | frontal cortex, normal |
| 9 | 92.1 | F | frontal cortex, normal |
| 11 | 79 | M | frontal cortex, severe AD |
| 12 | 73.6 | F | frontal cortex, severe AD |
| 14 | 82 | F | frontal cortex, severe AD |
| 17 | 72 | M | frontal cortex, severe AD |
| 19 | 76 | F | frontal cortex, severe AD |
|  | | | |
| PM032 | 68 | M | neural retina, normal |
| PM038 | 79 | M | neural retina, normal |
| PM039 | 82 | M | neural retina, normal |
| PM049 | 69 | F | neural retina, normal |
| PM028 | 86 | M | neural retina, AMD-affected |
| PM033 | 86 | M | neural retina, AMD-affected |
| PM040 | 80 | M | neural retina, AMD-affected |
| PM051 | 86 | F | neural retina, AMD-affected |
| PM030 | 76 | M | neural retina, normal, history of AD |
| PM041 | 77 | F | neural retina, normal, history of AD |
| PM045 | 81 | M | neural retina, normal, history of AD |

Alzheimer’s disease (AD) in frontal cortex was evaluated by Clinical Dementia Rating with no dementia representing normal brain and severe dementia representing severe AD brain. Retina status was assessed by fellowship trained vitreo-retinal specialist and information about AD was in the clinical history provided by the eye bank.
