# Supplementary material for: Quantification of Histone Deacetylase Isoforms in Human Frontal Cortex, Human Retina, and Mouse Brain
Source: PLoS One. 2015 May 11;10(5):e0126592. doi: 10.1371/journal.pone.0126592 (PMC4427357; doi:10.1371/journal.pone.0126592)
Supplement: S5 Table — (DOCX) [file pone.0126592.s008.docx]

**S5 Table.** **Results of one way ANOVA and Tukey post hoc analysis.**

| **Human neural retina** |  |
| --- | --- |
| HDAC1,2 |  |
| Between groups | p = 1.37x10^-9^ |
| Control vs AD | p < 0.01 |
| Control vs AMD | p < 0.01 |
| AD vs AMD | p < 0.01 |
|  |  |
| HDAC5 |  |
| Between groups | p = 1.2x10^-5^ |
| Control vs AD | p < 0.01 |
| Control vs AMD | p < 0.01 |
| AD vs AMD | p < 0.01 |
|  |  |
| HDAC6 |  |
| Between groups | p = 1.45x10^-15^ |
| Control vs AD | p < 0.01 |
| Control vs AMD | p < 0.01 |
| AD vs AMD | p < 0.01 |
|  |  |
| HDAC7 |  |
| Between groups | p = 0.00024 |
| Control vs AD | p < 0.01 |
| Control vs AMD | not significant |
| AD vs AMD | p < 0.01 |
|  |  |
| **HDAC profiles in control tissues** |  |
| HDAC1,2 |  |
| Between groups | p = 3.79x10^-7^ |
| Human frontal cortex vs human retina | p < 0.01 |
| Human frontal cortex vs mouse brain | not significant |
| Human retina vs mouse brain | p < 0.01 |
|  |  |
| HDAC5 |  |
| Between groups | p = 5.42x10^-6^ |
| Human frontal cortex vs human retina | p < 0.05 |
| Human frontal cortex vs mouse brain | p < 0.01 |
| Human retina vs mouse brain | p < 0.01 |
|  |  |
| HDAC6 |  |
| Between groups | p = 8.97x10^-28^ |
| Human frontal cortex vs human retina | p < 0.01 |
| Human frontal cortex vs mouse brain | p < 0.01 |
| Human retina vs mouse brain | p < 0.01 |

One way ANOVAs and Tukey HSD testing were performed using VassarStats (www.vassarstats.net) for each HDAC listed. For human neural retina, Tukey pairwise comparisons were performed to determine significance of changes in control to disease states, AD and AMD, and to also confirm that each measured HDAC in AD was significantly lower than in AMD. For the purpose of comparing HDAC profiles between different types of healthy tissues, Tukey pairwise comparisons were calculated. HDACs 3, 4, and 7 were not included in cross-tissue comparisons because they were only quantified in one tissue type.
