# Supplementary material for: Quantification of Histone Deacetylase Isoforms in Human Frontal Cortex, Human Retina, and Mouse Brain
Source: PLoS One. 2015 May 11;10(5):e0126592. doi: 10.1371/journal.pone.0126592 (PMC4427357; doi:10.1371/journal.pone.0126592)
Supplement: S6 Table — (DOCX) [file pone.0126592.s009.docx]

**S6 Table. Transitions used for quantification.**

|  |  |  |  | **Precursor (m/z)** | **Product Ions (m/z)** | | | |
| --- | --- | --- | --- | --- | --- | --- | --- | --- |
| **Isoform** | **Peptide** | **Species** |  |  | **1** | **2** | **3** |  |
| HDAC1,2 | YGEYFPGTGDLR | human/mouse | L | 687.8 | 715.4 (y_7_) | 862.4 (y_8_) | 1025.5 (y_9_) |  |
|  |  |  | H | 695.3 | 725.3 (y_7_) | 873.4 (y_8_) | 1037.5 (y_9_) |  |
| HDAC3 | YTGASLQGATQLNNK | human/mouse | L | 783.4 | 845.4 (y_8_) | 973.5 (y_9_) | 1173.6 (y_11_) |  |
|  |  |  | H | 793.4 | 857.4 (y_8_) | 987.5 (y_9_) | 1189.6 (y_11_) |  |
| HDAC4 | ESAVASTEVK | human/mouse | L | 510.8 | 563.3 (y_5_) | 634.3 (y_6_) | 733.4 (y_7_) |  |
|  |  |  | H | 516.2 | 569.3 (y_5_) | 641.3 (y_6_) | 741.4 (y_7_) |  |
|  | DQPVELLNPAR | human/mouse | L | 626.3 | 683.4 (y_6_) | 812.5 (y_7_) | 1008.6 (y_9_) |  |
|  |  |  | H | 634.3 | 693.4 (y_6_) | 823.4 (y_7_) | 1021.5 (y_9_) |  |
| HDAC5 | LSTQQEAER | human/mouse | L | 510.8 | 632.3 (y_5_) | 760.4 (y_6_) | 861.4 (y_7_) |  |
|  |  |  | H | 531.3 | 641.3 (y_5_) | 771.3 (y_6_) | 873.4 (y_7_) |  |
|  | GALVGSVDPTLR | human | L | 592.8 | 601.3 (y_5_) | 787.4 (y_7_) | 844.5 (y_8_) |  |
|  |  |  | H | 600.3 | 609.3 (y_5_) | 797.4 (y_7_) | 855.4 (y_8_) |  |
| HDAC6 | EQLIQEGLLDR | human/mouse | L | 657.4 | 702.4 (y_6_) | 830.4 (y_7_) | 943.5 (y_8_) |  |
|  |  |  | H | 665.3 | 711.4 (y_6_) | 841.4 (y_7_) | 955.5 (y_8_) |  |
|  | LEELGLAGR | human | L | 479.3 | 586.4 (y_6_) | 715.4 (y_7_) | 844.5 (y_8_) |  |
|  |  |  | H | 485.3 | 595.3 (y_6_) | 725.4 (y_7_) | 855.4 (y_8_) |  |
| HDAC7 | TLEPLETEGATR | human | L | 658.8 | 763.4 (y_7_) | 876.4 (y_8_) | 1102.5 (y_10_) |  |
|  |  |  | H | 666.3 | 773.3 (y_7_) | 887.4 (y_8_) | 1115.5 (y_10_) |  |

Transitions are listed for both unlabeled, light (L) and fully ^15^N-labeled, heavy (H) peptides. All precursor ions were +2 charge and product ions were +1 charge. Additionally, y-ion information is included for reference.
