# Supplementary material for: Quantification of Histone Deacetylase Isoforms in Human Frontal Cortex, Human Retina, and Mouse Brain
Source: PLoS One. 2015 May 11;10(5):e0126592. doi: 10.1371/journal.pone.0126592 (PMC4427357; doi:10.1371/journal.pone.0126592)
Supplement: S7 Table — (DOCX) [file pone.0126592.s010.docx]

**S7 Table. Comparison of individual Q-peptide and protein measurements.**

| Mouse brain (control) | pmol/mg |
| --- | --- |
| ESAVASTEVK | 0.229 ± 0.028 |
| DQPVELLNPAR | 0.231 ± 0.034 |
| **HDAC4 (total)** | **0.230 ± 0.029** |
|  |  |
| Human frontal cortex (control) | pmol/mg |
| GALVGSVDPTLR | 0.085 ± 0.020 |
| LSTQQEAER | 0.080 ± 0.010 |
| **HDAC5 (total)** | **0.083 ± 0.016** |
|  |  |
| EQLIQEGLLDR | 0.102 ± 0.010 |
| LEELGLAGR | 0.112 ± 0.018 |
| **HDAC6 (total)** | **0.106 ± 0.015** |

Three transitions for each Q-peptide (Supporting Information S5 Table) were used for measurements in biological replicate samples of control human frontal cortex (n=5) and whole hemispheres of control mice (n=2). For HDACs 4, 5, and 6, two Q-peptides were used for quantification of each protein. Concentrations of mean ± SD for individual peptides are presented for comparison to mean ± SD for protein measurements, which include a total of both peptides (bold). Student t-test was performed and concentrations of peptides for the same protein were not statistically different in comparison to each other, indicating a consensus in peptide measurements.
